# Supplementary material for: Climate-Driven Adaptive Differentiation in Melia azedarach: Evidence from a Common Garden Experiment
Source: Genes (Basel). 2022 Oct 22;13(11):1924. doi: 10.3390/genes13111924 (PMC9689450; doi:10.3390/genes13111924)
Supplement: Supplementary file 1 [file genes-13-01924-s001.zip › Table S1. The information of 15 pair primers for SSR analysis .pdf]

Table S1. The information of 15 pair primers for SSR analysis

Locus code	Primer sequence（5'-3'）	Tm (℃)	Repeat motif	Expected allele size range(bp)	Number of alleles	Genbank accession no.
SSR2	F: GATAGCGGAGCCGGTGATT<br>R: GGATGGAAGGCTCAAGATTCG	60	(AG)12<br>TT(AG)7	242-284	21	HM041034
SSR29	F: ATGGATGAGTGTGCGATAGG<br>R: TGTGATGTAGGAGTCTGAAC	60	(TC)7	168-184	9	DQ453912
SSR54	F: GAGAAGAGAAGGCTGTGTGC<br>R: GAAACCTGATTCGTCGTCGT	62	(CT)47	146-170	14	GQ254833
SSR59	F: CTCCTCGTTTGCCACTCATT<br>R: AAACAGAGGGTTTTCGGTGC	56	(CT)20	196-330	25	GQ254847
SSR74	F: GTCCACGCAAACAGAGACAC<br>R: TTGGCTTGGCTTTCTCTTTC	58	(CT)10	204-240	14	FM161912
SSR111	F: CCCTATTCATTGTCCCTCCA<br>R: GTCCTCCTGGAATTCTGTGC	60	(CT)19	126-164	20	AB674473
SSR113	F: CAACCATGGTGTCGAGAAGA<br>R: TGCTTAATTTGCCTGTGCAT	60	(AG)23	168-192	11	AB674475
SSR114	F:CTAGACCAGCCCCAAGAACA<br>R: TTCAAGGGCTTCTTCTGAATC	60	(AC)9	204-232	15	AB674476
SSR116	F: GGAACCCCAATTTAGGAACT<br>R: TGCTTGGTGAAAACCATAGA	58	(AC)11	180-214	18	AB674478
SSR117	F: CCGTGTAAGAGTGCCAAATC<br>R: TCTTGGAGGTGAGATCAAGTG	60	(TA)5(TG)17	86-188	10	AB674479
SSR118	F:TCTGTTGGTGGTGTTGTCAC<br>R:TAATGTGGATGCAAGCAGTG	58	(TC)22(AC)1<br>0	148-182	18	AB674480
SSR119	F: CAAGCACACACAAGGATTTG<br>R: TGGCAACTCTCAGGTATCAA	52	(GA)21	200-236	18	AB674481
SSR120	F: GTTGTTTGTGGGTGTGTGTT<br>R: GAGAGAGAACCCAAAGGAAAA	60	(TC)7(AC)12	260-312	27	AB674482
SSR122	F: GTGCAGTGTCCATGTTGAAG<br>R: GACATTTTCTCTGCAAGGTCA	60	(TG)14	158-260	32	AB674484
SSR123	F: TTCCCTCAGCATTAAGGTGT<br>R: CAGGCAAAGGAAGGTAGGTA	60	(TA)9(TG)8	84-112	8	AB674485
