# Supplementary material for: Climate-Driven Adaptive Differentiation in Melia azedarach: Evidence from a Common Garden Experiment
Source: Genes (Basel). 2022 Oct 22;13(11):1924. doi: 10.3390/genes13111924 (PMC9689450; doi:10.3390/genes13111924)
Supplement: Supplementary file 1 [file genes-13-01924-s001.zip › Table S2. Information and source of the 135 primers.pdf]

Table S2. Information and source of the 135 primers

Code number	NCBI Number	Primer F'	Primer R'	Source	Reference
1	HM041033	GCATGAGCTTGAGAGAATC	CAGAGGACTGAAGTAGCTGA	*Swietenia macrophylla*	(Lemes,2011)
2	HM041034	GATAGCGGAGCCGGTGATT	GGATGGAAGGCTCAAGATTCG	*Swietenia macrophylla*	(Lemes,2011)
3	HM041035	TTCCTCTTCCTTGACCGCTC	CGTACGGTTATGATCAGCGAC	*Swietenia macrophylla*	(Lemes,2011)
4	HM041038	AGAGTGTTCGAGAGCCTCAA	AGAGCCGAATTCACCGAT	*Swietenia macrophylla*	(Lemes,2011)
5	HM041039	CTGTCATGCATATCGTTGGA	GGGCAGATAAAGAGGAACAAG	*Swietenia macrophylla*	(Lemes,2011)
6	HM041046	CACATCGTGAGGAATTTACC	TGGGATTTGTGTTCACCTT	*Swietenia macrophylla*	(Lemes,2011)
7	HM041040	GCTCGGTGGTGTTACAGTT	CAGTATGACAGTATCAAGGGGA	*Swietenia macrophylla*	(Lemes,2011)
8	HM041041	CGTGGTTGCTACCTATATGC	TATACCGACCGCGTTAAGT	*Swietenia macrophylla*	(Lemes,2011)
9	HM041042	CAGTCATGGAGCGTAGCTAA	TGCAGTTTCAGAACCTGAATC	*Swietenia macrophylla*	(Lemes,2011)
10	HM041043	TAGGAACCAACCACCAAC	GTTCTCCTGCTCTCTTTGA	*Swietenia macrophylla*	(Lemes,2011)
11	HM041044	TCAGGAATGGAAGGTACAGG	CAGTCATGGAGCGTAGCTAA	*Swietenia macrophylla*	(Lemes,2011)
12	HM041045	GAACTGGCAATGTGCTGACT	TCGGCAATAGCAAGACATTC	*Swietenia macrophylla*	(Lemes,2011)
13	---	ACACATATGAATAGATATGCAGAGA	CACATAAAGCAACAGATACGCCAGC	*Carapa guianensis*	(Dayanandan,1999)
14	---	TGCATTTCTTCTCCTTGCTTCTGG	TCTTGTACGTTGGCTCTTGCACATTC	*Carapa guianensis*	(Dayanandan,1999)
15	---	ACATTCTCTCTCTTTTTCTCTCAGC	TAGTGAAAAAGACTAGACCTCACAGT	*Carapa guianensis*	(Dayanandan,1999)
16	---	AAGACTTCCCCCAGATTTGTTTTT	TGTACCAGTTCAGTTGATGCTCA	*Carapa guianensis*	(Dayanandan,1999)
17	---	GTGGCGTAACAGACCAAAAC	CCAGAGATACTCCATTCCAG	*Toona ciliata var*. *pubescens*	(Liu, 2006)
18	---	CACTTAGCCTGTAGCACTAG	CTTCACCAAACTCATCTCTC	*Toona ciliata var*. *pubescens*	(Liu, 2006)
19	---	AGTAATAGCCTGTAGAGCAG	AGAGTGGGGTGGTCGATGAG	*Toona ciliata var*. *pubescens*	(Liu, 2006)
20	---	GAAACCAGCAGGCAGAGC	GAAGAAGGGTGAGCGAGA	*Toona ciliata var*. *pubescens*	(Liu, 2006)
21	---	CCTGAGATTCGTCCAAGT	ACCGCATTAGTACCAGTAG	*Toona ciliata var*. *pubescens*	(Liu, 2006)
22	---	GATTACGCCAGGCAAACG	TTGAATATGGGAGAAGGT	*Toona ciliata var*. *pubescens*	(Liu, 2006)

Table S2. to be continued

23	---	ATTAGCTTACCGCATTAG	CAAGGCGGCACTTGTTTT	*Toona ciliata var*. *pubescens*	(Liu, 2006)
24	---	GGCGACTAATGAGTGAGA	AGAACGCAGCGAACTACA	*Toona ciliata var*. *pubescens*	(Liu, 2006)
25	---	GTGTTAGCCGACGAAGAA	CATCAATTACAGTTGGTGGTA	*Toona ciliata var*. *pubescens*	(Liu, 2006)
26	---	AAGCCAGTCAGCAACCTA	GATTAAGTAATATTGGGTGGT	*Toona ciliata var*. *pubescens*	(Liu, 2006)
27	---	TGTCTCAGTTTATGCTGGCGT	CTGCCCAATCAACAAGAG	*Toona ciliata var*. *pubescens*	(Liu, 2006)
28	---	AACGGCTCAAATTCTATA	CATTACTACCAGTATCCA	*Toona ciliata var*. *pubescens*	(Liu, 2006)
29	---	ATGGATGAGTGTGCGATAGG	TGTGATGTAGGAGTCTGAAC	*Toona ciliata var*. *pubescens*	(Liu, 2006)
30	---	TTAGTACCAGCAGCTTACC	AAGCCTGTCCACTTTCTC	*Toona ciliata var*. *pubescens*	(Liu, 2006)
31	---	TCGACGATTATGATGACCATG	TGCAGTCTCCCTACTCAA	*Toona ciliata var*. *pubescens*	(Liu, 2006)
32	---	GCTTAGCTTACCGCATTAGT	ATTACGCCAGGACTTTTCAC	*Toona ciliata var*. *pubescens*	(Liu, 2006)
33	---	GGTGGACTTAGCAGGTAG	AATCTCAGAGGAAAGGGAC	*Toona ciliata var*. *pubescens*	(Liu, 2006)
34	---	CATCGGGAGGTTTAGGTC	GTGTTCATGGGAGTAGGG	*Toona ciliata var*. *pubescens*	(Liu, 2006)
35	---	TCCCTTCCAAGCTTGTTCCTG	TGGCCCTTTCTCGATGTC	*Swietenia macrophylla*	(Cespedes,2003)
36	AJ000405	AGGGTCTTTGTTGATGAATACC	ATCATCGCCGTTGATACATT	*Swietenia macrophylla*	(Cespedes,2003)
37	AJ000407	GCGATGAATGTTGTACACCG	AGCAGCCCGGTACTAGCATGT	*Swietenia macrophylla*	(Cespedes,2003)
38	AJ000410	TGGAGTAAAGTCGAGGGCTG	GGCTGGATATGGCACTTGTT	*Swietenia macrophylla*	(Cespedes,2003)
39	AJ000389	CCTGAAAGCTTAATGGCCAC	TCAGCCTTTGAGGGATGAAC	*Swietenia macrophylla*	(Cespedes,2003)
40	AF428115	GCGCGATTGATTGACTTC	GCGCTTAGCATTATTCTCC	*Khaya senegalensis*	(Sexton,2010)
41	HQ200180	CAAAATATTTAACTAGCCATCCA	CTCAAGTCCAATTCAATTCAT	*Khaya senegalensis*	(Sexton,2010)
42	HQ200181	ACCATGCAATGCTAACTTTAT	CTTTTGTGTCCAAAAATTGAC	*Khaya senegalensis*	(Sexton,2010)
43	HQ200182	CTTAAGACATTTGACCAATCC	GTCGGACAATTGTGTTTTAGT	*Khaya senegalensis*	(Sexton,2010)
44	HQ200183	CACTCAGACGCAATAAATTAAA	GCGATTTTTATATCTGGTTTTC	*Khaya senegalensis*	(Sexton,2010)
45	HQ200184	ATCCAAAACGCTTCTAAAGTC	GGATGTTATGGTGAAAAATTG	*Khaya senegalensis*	(Sexton,2010)
46	HQ200185	CAATATAAGGGACAATACTCTCA	CAACATAGATCCATCGTGAGT	*Khaya senegalensis*	(Sexton,2010)
47	HQ200186	TGGGGTAGTTTTAAAGTTGTG	AAGGTTGGAAGAGTAATTTGC	*Khaya senegalensis*	(Sexton,2010)

Table S2. to be continued

48	HQ200187	TATAAGTGAATAAACCGCGTA	TATAGCCTATCTTGTCGCAAA	*Khaya senegalensis*	(Sexton,2010)
49	HQ200188	AAACGTGCTGGCTAGTTATTA	TGTAAGTCAACAGCAAGAAAA	*Khaya senegalensis*	(Sexton,2010)
50	HQ200189	TTTCAACTCTTCAATCTTCATCT	GGCACTACCAATATTTTGTTTT	*Khaya senegalensis*	(Sexton,2010)
51	HQ200190	ACAACTCTTTTACGTCCACCT	CATCATCTTCTTCTGTTACGG	*Khaya senegalensis*	(Sexton,2010)
52	GQ254820	TCTACTGGTTATGGCTGTGC	TGGTTGTCGTGTAGTGCAAA	*Cabralea canjerana*	(Pereira,2010)
53	GQ254827	CTCAGCAGCAACAAGAGTA	CAATGGTCGGACATCTTCCC	*Cabralea canjerana*	(Pereira,2010)
54	GQ254833	GAGAAGAGAAGGCTGTGTGC	GAAACCTGATTCGTCGTCGT	*Cabralea canjerana*	(Pereira,2010)
55	GQ254834	TGGTCCGAGGATCAAAACA	TGGGGACGAAATGGAACTTG	*Cabralea canjerana*	(Pereira,2010)
56	GQ254838	GCCAAGATCCAGCTAATGCT	GCTGTTTGCCTTCCCTAACT	*Cabralea canjerana*	(Pereira,2010)
57	GQ254841	GTGACAGAGCGAGATTCCAT	CCCCTTCAGCTCCAAAATGT	*Cabralea canjerana*	(Pereira,2010)
58	GQ254845	CTTGCGATGGCAATCAAACC	AGAAGGGCATACCAGGTTCT	*Cabralea canjerana*	(Pereira,2010)
59	GQ254847	CTCCTCGTTTGCCACTCATT	AAACAGAGGGTTTTCGGTGC	*Cabralea canjerana*	(Pereira,2010)
60	AJ000404	ATGAACCATGTTTCAATCTCA	GGGAGCATACATGATATGTACA	*Swietenia humilis*	(White and Powell,1997a)
61	---	TTCCTCATTTCACTGTCATTACC	ATTATTACTGCAACAATCTGCAGGCT	*Swietenia humilis*	(White and Powell,1997a)
62	AJ000406	GCAATGTTGAGGAACCGC	AGCTTGGATTCTCCCATGC	*Swietenia humilis*	(White and Powell,1997a)
63	AJ000408	TGTTCCATGAGTTTACACAGCT	ATTGTTTTGCATTCAGGTTTTT	*Swietenia humilis*	(White and Powell,1997a)
64	AJ000409	AAACTTAAGGCAGCCACAGC	ACAGTGGCTCTGCTGGTTTT	*Swietenia humilis*	(White and Powell,1997a)
65	DQ778303	TCAATGCAATTTAGGAGGAA	TGCTTGTTGAACCCTGTG	*Toona ciliata var．pubescens*	(Liu, 2007)
66	DQ453903	GACTCGTGACACTTAGCCTGTA	CTGGCGTAATCATGGTCATAC	*Toona ciliata var．pubescens*	(Liu, 2007)
67	DQ453904	TAGGAAAGGCAAGGTGGG	GGGTGGTCGATGAGGGTT	*Toona ciliata var．pubescens*	(Liu, 2007)

Table S2. to be continued

68	---	GCCTGAGATTCGTCCAAGT	ACCGCATTAGTACCAGTAG	*Toona ciliata var．pubescens*	(Liu, 2007)
69	---	TCGACGATTATGACCATG	TGCAGTCTCCCTACTCAA	*Toona ciliata var．pubescens*	(Liu, 2007)
70	FM161907	TGGTAACCAATCTGTGTGTGC	CGGTTCCTGGTTTCTTTTGG	*Azadirachta indica*	(Boontong,2009)
71	FM161908	GAAAGGAGGGTTTCAAATCA	TCGGCCGAACACAATTTTA	*Azadirachta indica*	(Boontong,2009)
72	FM161909	ACAAAATTTTTCCCGTCGAG	AGAGCTATGAATGGTGGACTCAC	*Azadirachta indica*	(Boontong,2009)
73	FM161911	CCACAAACAAATGGGAAACC	CCCTTATTACAAAAGAAGAGGGAAG	*Azadirachta indica*	(Boontong,2009)
74	FM161912	GTCCACGCAAACAGAGACAC	TTGGCTTGGCTTTCTCTTTC	*Azadirachta indica*	(Boontong,2009)
75	FM161913	ATTTGTGTGTGCGTGCTAGG	CGAGGAACTGAGACRCCRGAA	*Azadirachta indica*	(Boontong,2009)
76	FM161914	TCCCAGTTATTCAACGTAGGC	TCTTAATCATGGATTGCTTCACA	*Azadirachta indica*	(Boontong,2009)
77	EF413962	TTTGCTTTGAGAAACCTTGT	AACTTTCGAATTGGTTAAGG	*Cedrela odorata*	(Hernández,2008)
78	EF413963	CAAAGACCAAGATTTGATGC	ACTATGGGTGGCACAACTAC	*Cedrela odorata*	(Hernández,2008)
79	EF413964	TCATTCTTGGATCCTGCTAT	GTGGGAAAGATTGTGAAGAA	*Cedrela odorata*	(Hernández,2008)
80	EF413965	ACTCCATTAACTGCCATGAA	ATTTTCATTCCCTTTTAGCC	*Cedrela odorata*	(Hernández,2008)
81	EF413966	GATCTCACCCACTTGAAAAA	GCTCATATTTGAGAGGCATT	*Cedrela odorata*	(Hernández,2008)
82	EF413967	CAATCAAACCAAAAATGGAT	GCAAATTAACCAGAAAAACG	*Cedrela odorata*	(Hernández,2008)
83	EF413968	GAGTGAGAAGAAGAATCGTGATAGC	GAGGTTCGATCAGGTCTTGG	*Cedrela odorata*	(Hernández,2008)
84	EF413969	ATTTTCATTCCCTTTTAGCC	TTATCATCTCCCTCACTCCA	*Cedrela odorata*	(Hernández,2008)
85	EF413970	CTCGTAATAATCCCATTCCA	GGAGATATTTTTGGGGTTTT	*Cedrela odorata*	(Hernández,2008)
86	JF423305	ACACTACCTCCACTGGCTC	AGATGGGACCGTGTACCTG	*African mahogany*	(KARAN,2012)
87	JF423306	ATGCAGCAACAAACAGCAG	ATCAGGAAGCTCGGGTTGC	*African mahogany*	(KARAN,2012)
88	JF423307	CAGCGAGCTATAATAAAGACCAAAG	ACCTGATGACTTCTCTCTCTCTC	*African mahogany*	(KARAN,2012)
89	JF423308	CGGCTTAGAGGTCAAAATCGG	TGCAACTTATGTACGTGAGAAAC	*African mahogany*	(KARAN,2012)
90	JF423309	GCAACAATGCACACAGGTC	ATAGACGCATACTCCGCCC	*African mahogany*	(KARAN,2012)
91	JF423310	GCAATGTAATACTTAAAAGCAGGTG	ATTTGTGAGGCGAGTGAAAG	*African mahogany*	(KARAN,2012)
92	JF423311	TGCAGACTTCTGTTGCTCC	TGGCGGTAGATGTGTGGTC	*African mahogany*	(KARAN,2012)

Table S2. to be continued

93	JF423312	TGTTCACTGGTCGGATGTG	GTTGGTGGCGATATGCTCG	*African mahogany*	(KARAN,2012)
94	JF423313	TTGAGGGAGGAGCAAGACG	TCTGGCCAATACCAAGTATCAC	*African mahogany*	(KARAN,2012)
95	JF423314	TTGGCAGTTACAAGCACGC	TGTGAGGTAGATTGAAGGGGAG	*African mahogany*	(KARAN,2012)
96	JF423315	AAGCATCAGCAGAAGCACC	TGGGATGTAACCGAGGTGTG	*African mahogany*	(KARAN,2012)
97	AJ000402	GAGATACAGTTGGTGGTTAGAGG	TCTTCACCTGTTTGCCTCTC	*Swietenia humilis*	(White and Powell,1997b)
98	AJ000411	TCATTTGGAATCTGGGTTCT	TTATTTCGTACCCTTCGCTC	*Swietenia humilis*	(White and Powell,1997b)
99	AJ000403	ATAAGCCAGATGACGGAACG	GCATACGGCTTTCTGGATGT	*Swietenia humilis*	(White and Powell,1997b)
100	FM161910	GCATCAGTCAGCCATAGTGC	TTGAAAAATCCTGGCGAGTG	*Azadirachta indica*	(Boontong,2009)
101	AF428116	TCTGCTACAGAGCTGGATGC	GTATGCTCGAAGAAGTCGTTG	*Swietenia macrophylla*	(Lemes,2002)
102	AF428117	CTTCTAATGTTCTGATGCCTG	AGCAACTCGTGAGGAATTTAC	*Swietenia macrophylla*	(Lemes,2002)
103	AF428118	CACCTTATGTACACCACACAG	GAAGGAGACACCAGCAATC	*Swietenia macrophylla*	(Lemes,2002)
104	AF428119	GCACTCAAGGTACACTATGAT	TACGTGTGAATGCGTCTAT	*Swietenia macrophylla*	(Lemes,2002)
105	AF428120	TGCTACTGTCAAGAGTGTAT	GACAAACATGTACCACAAG	*Swietenia macrophylla*	(Lemes,2002)
106	AF428121	CCTTATGTTCACCACACAGTA	GAGACACCAGCAATCCAG	*Swietenia macrophylla*	(Lemes,2002)
107	AF428122	GCAGTACTCGCCTATCTTCA	TGAGAACTGCAGAATCCTTT	*Swietenia macrophylla*	(Lemes,2002)
108	AF428123	GCCATTGGTCTCAATCTTAC	GGAAGAGTCTTAGAACACAG	*Swietenia macrophylla*	(Lemes,2002)
109	AF428124	GCAATTTCCAGAAGAAACC	CTGTAGGCGATAACAATCAG	*Swietenia macrophylla*	(Lemes,2002)
110	AB674472	TGTCTATCCGGTGAAGGTGT	TTGCATACAATAATATCGTGCAAC	*Melia volkensii*	(Hanaoka,2012)
111	AB674473	CCCTATTCATTGTCCCTCCA	GTCCTCCTGGAATTCTGTGC	*Melia volkensii*	(Hanaoka,2012)
112	AB674474	AGAGAAGACAGATCCCCCAGT	CAACACAACACAACACAGCAA	*Melia volkensii*	(Hanaoka,2012)
113	AB674475	CAACCATGGTGTCGAGAAGA	TGCTTAATTTGCCTGTGCAT	*Melia volkensii*	(Hanaoka,2012)
114	AB674476	CTAGACCAGCCCCAAGAACA	TTCAAGGGCTTCTTCTGAATC	*Melia volkensii*	(Hanaoka,2012)

Table S2. to be continued

115	AB674477	CCAATGTTGTTCAACTATATGAGGTC	GAATTTCGAAGAGTGCCAAAA	*Melia volkensii*	(Hanaoka,2012)
116	AB674478	GGAACCCCAATTTAGGAACT	TGCTTGGTGAAAACCATAGA	*Melia volkensii*	(Hanaoka,2012)
117	AB674479	CCGTGTAAGAGTGCCAAATC	TCTTGGAGGTGAGATCAAGTG	*Melia volkensii*	(Hanaoka,2012)
118	AB674480	TCTGTTGGTGGTGTTGTCAC	TAATGTGGATGCAAGCAGTG	*Melia volkensii*	(Hanaoka,2012)
119	AB674481	CAAGCACACACAAGGATTTG	TGGCAACTCTCAGGTATCAA	*Melia volkensii*	(Hanaoka,2012)
120	AB674482	GTTGTTTGTGGGTGTGTGTT	GAGAGAGAACCCAAAGGAAAA	*Melia volkensii*	(Hanaoka,2012)
121	AB674483	CCTTGTCGTGTTAAGTGTCG	TACTCATTTGGGGGTTGCTA	*Melia volkensii*	(Hanaoka,2012)
122	AB674484	GTGCAGTGTCCATGTTGAAG	GACATTTTCTCTGCAAGGTCA	*Melia volkensii*	(Hanaoka,2012)
123	AB674485	TTCCCTCAGCATTAAGGTGT	CAGGCAAAGGAAGGTAGGTA	*Melia volkensii*	(Hanaoka,2012)
124	AB674486	ACTTTGTCGCCATATCGAAG	CTCCACTCACTTCTCGGTGT	*Melia volkensii*	(Hanaoka,2012)
125	BV725398	TTGCTTACGCGTGGATATTG	CAGCACCTTGTTTCGCATTA	*Guarea guidonia*	(de Lima,2009)
126	BV725397	TGAAATTTCGGCTCTTGCTT	ATTCTTTTGCTTACGCGTGG	*Guarea guidonia*	(de Lima,2009)
127	BV725399	TAGGCAGTTGGGAAGGCTTA	GATCCGGAACTTACCTGCAA	*Guarea guidonia*	(de Lima,2009)
128	BV725400	TTTTATGGATGCGTAAAGGATG	TTGGTTATGTGGCGATTTTG	*Guarea guidonia*	(de Lima,2009)
129	BV725401	TCTTGCTTACGCGTGGACTA	TACGCGTGGACTAACAGACG	*Guarea guidonia*	(de Lima,2009)
130	BV725392	AGCCTTGTCGCCAAAACTAA	ATCACACCTATGTGGGCTGC	*Guarea guidonia*	(de Lima,2009)
131	BV725393	ATTCTCTTGCTTACGCGTGG	CCGGAACTAACCTGCAAAAC	*Guarea guidonia*	(de Lima,2009)
132	BV725394	GCGGATATGATTCGAGAGGT	TCTTGCTTACGCGTGGACTA	*Guarea guidonia*	(de Lima,2009)
133	BV725395	CATATGGCAAGGTGCTGATG	AAATGTGGTTGCGTGTGTGT	*Guarea guidonia*	(de Lima,2009)
134	BV725396	ATTCTCTTGCTTACGCGTGG	GAGGAGCCTGATCACCAGAA	*Guarea guidonia*	(de Lima,2009)
135	BV725402	TCTTGCTTACGCGTGGACTA	GTGGAGGAGCCTGGTCACTA	*Guarea guidonia*	(de Lima,2009)
