# Supplementary material for: Climate-Driven Adaptive Differentiation in Melia azedarach: Evidence from a Common Garden Experiment
Source: Genes (Basel). 2022 Oct 22;13(11):1924. doi: 10.3390/genes13111924 (PMC9689450; doi:10.3390/genes13111924)
Supplement: Supplementary file 1 [file genes-13-01924-s001.zip › Table S3. Tests of Hardy-Weinberg equilibrium at each SSR locus in each population.pdf]

Table S3. Tests of Hardy-Weinberg equilibrium at each SSR locus in each population.

population	SSR02	SSR29	SSR54	SSR59	SSR74	SSR111	SSR113	SSR114	SSR116	SSR117	SSR118	SSR119	SSR120	SSR122	SSR123
412	0.0175	0.0255	0.1715	0.2278	0.0138	0.0128	0.0009	0.0007	0.6967	0.3675	0.0066	0.0000	0.9856	0.0000	0.0390
	0.0055	–	0.0147	0.0286	0.0036	0.0045	0.0004	0.0006	0.0207	0.0084	0.0022	0.0000	0.0057	0.0000	0.0053
741	0.0000	0.0188	0.5966	0.1177	0.0490	0.1590	0.0236	0.0057	0.0936	0.2941	0.0000	0.0036	0.0000	0.0346	0.6654
	0.0000	–	0.0179	0.0092	0.0064	0.0167	0.0033	0.0009	0.0105	–	0.0000	0.0036	0.0000	0.0077	0.0076
631	0.0011	0.0015	0.0381	0.3549	0.0549	0.0015	0.0004	0.0598	0.0499	0.0009	0.0037	0.0000	0.0000	0.0072	0.1038
	0.0010	–	0.0046	0.0188	0.0062	0.0007	–	0.0044	0.0044	–	0.0014	0.0000	0.0000	0.0043	–
739	0.0014	0.1336	0.0003	0.0147	0.0064	0.0145	0.0012	0.8614	0.0045	0.0000	0.0000	0.0000	0.0000	0.0000	0.1532
	0.0010	0.0049	0.0003	0.0042	0.0018	0.0036	–	0.0217	0.0015	0.0000	0.0000	0.0000	0.0000	0.0000	–
205	0.0019	0.0019	0.0383	0.1095	0.0020	0.9894	0.0000	0.0193	0.2185	0.0817	0.0292	0.0000	0.0666	0.0017	0.5650
	0.0013	–	0.0041	0.0145	–	0.0029	0.0000	0.0052	0.0106	0.0056	0.0058	0.0000	0.0130	0.0006	0.0085
524	0.0000	0.0026	0.0012	0.0111	0.2966	0.3994	0.0012	0.0002	0.0013	0.0009	0.0000	0.0000	0.0000	0.0000	0.2121
	0.0000	0.0010	0.0006	0.0038	0.0141	0.0315	–	0.0002	–	–	0.0000	0.0000	0.0000	0.0000	0.0076
525	0.0201	0.0000	0.0005	0.4280	0.0015	0.0000	0.0010	0.0000	0.0008	0.0013	0.0017	0.0055	0.0000	0.0000	0.8018
	–	–	0.0003	0.0291	–	0.0000	–	0.0000	–	–	0.0012	0.0041	0.0000	–	0.0058
628	0.0034	0.0020	0.7983	0.0000	0.0005	0.1318	0.0000	0.0034	0.1072	0.0643	0.0019	0.0000	0.1214	0.0000	0.0610
	0.0012	–	0.0133	0.0000	0.0004	0.0156	0.0000	–	0.0168	0.0040	0.0015	0.0000	0.0155	0.0000	0.0082
415	0.0008	0.0008	0.1531	0.4042	0.0012	0.0006	0.0000	0.0016	0.0109	0.0023	0.0004	0.0005	0.0000	0.0000	0.0964
	0.0008	–	0.0143	0.0158	–	0.0004	0.0000	–	0.0054	0.0009	0.0004	0.0004	0.0000	0.0000	0.0056
307	0.0014	0.0020	0.2731	0.1964	0.0010	0.0139	0.0178	0.0005	0.2327	0.6960	0.0864	0.0000	0.0000	0.0125	0.0878
	0.0014	–	0.0237	0.0175	–	0.0031	0.0023	–	0.0097	0.0109	0.0161	0.0000	0.0000	0.0065	0.0040
842	0.0000	0.1304	0.0006	0.0002	0.0024	0.1369	0.0000	0.0010	0.0017	0.0100	0.0005	0.0000	0.0000	0.0000	0.0002
	0.0000	0.0151	0.0005	0.0001	–	0.0108	–	0.0009	–	0.0034	–	0.0000	0.0000	0.0000	–
308	0.0127	0.0278	0.1556	0.2030	0.2117	0.5165	0.0000	0.0017	0.0738	0.0024	0.0000	0.0000	0.5910	0.0000	0.4162
	0.0033	0.0030	0.0116	0.0414	0.0147	0.0300	0.0000	0.0012	0.0071	0.0006	0.0000	0.0000	0.0447	0.0000	0.0069

Table S3 to be continued

310	0.0041	0.0218	0.8047	0.0581	0.0015	0.7327	0.0041	0.1003	0.4607	0.1775	0.0006	0.0000	0.0000	0.0000	0.0098
	0.0021	0.0030	0.0146	0.0105	0.0009	0.0259	0.0014	0.0090	0.0215	0.0109	0.0006	0.0000	0.0000	0.0000	0.0026
843	0.0006	0.0065	0.2671	0.6800	0.0032	0.4485	0.0001	0.0000	0.6065	0.0126	0.0005	0.1265	0.0023	0.0000	0.0542
	0.0004	0.0015	0.0145	0.0382	0.0009	0.0427	–	0.0000	0.0342	0.0023	0.0003	0.0221	0.0016	0.0000	0.0032
102	0.0000	0.0009	0.0593	0.0082	0.0032	0.0327	0.0005	0.0000	0.0789	0.0125	0.0000	0.0000	0.0000	0.0000	0.1457
	0.0000	–	0.0136	0.0014	–	0.0067	–	0.0000	0.0160	0.0028	0.0000	0.0000	0.0000	0.0000	0.0105
844	0.0067	0.0154	0.2220	0.2343	0.0073	0.7128	0.0003	0.0080	0.0141	0.0706	0.0018	0.0000	0.0004	0.0000	0.0323
	0.0033	–	0.0104	0.0253	0.0018	0.0156	–	0.0036	0.0026	0.0049	0.0009	0.0000	0.0004	0.0000	0.0025
1061	0.0000	0.0138	0.2520	0.0026	0.0004	0.5005	0.0007	0.0024	0.0439	0.0049	0.0829	0.0000	0.2527	0.0000	0.0014
	0.0000	0.0027	0.0124	0.0008	–	0.0143	0.0004	0.0012	0.0144	0.0021	0.0123	0.0000	0.0251	0.0000	0.0005
1767	0.0049	0.0001	0.0282	0.0446	0.0163	0.4580	0.0189	0.0031	0.0147	0.0006	0.0008	0.0000	0.0023	0.0000	0.6935
	0.0026	0.0001	0.0038	0.0057	–	0.0275	–	–	0.0018	0.0005	0.0006	0.0000	0.0014	0.0000	0.0100
754	0.0158	0.0150	0.0009	0.0000	0.0083	0.0407	0.0000	0.3577	0.0046	0.0486	0.0012	0.0000	0.1079	0.0000	0.1000
	0.0026	0.0017	–	0.0000	0.0012	0.0036	0.0000	0.0145	0.0013	0.0044	0.0012	0.0000	0.0190	0.0000	–
959	0.0892	0.0040	0.0000	0.0000	0.0569	0.0138	0.0000	0.0000	0.0001	0.0000	0.0001	0.0000	0.0000	0.0000	0.0003
	0.0116	–	0.0000	0.0000	0.0038	0.0021	0.0000	0.0000	0.0001	0.0000	0.0001	0.0000	0.0000	0.0000	–
1565	0.0136	0.0057	0.1847	0.0011	0.0345	0.0672	0.0006	0.0038	0.0011	0.0137	0.0051	0.0012	0.0000	0.0000	0.1214
	0.0021	0.0011	0.0096	0.0007	0.0038	0.0130	–	0.0028	0.0009	0.0027	0.0023	0.0012	0.0000	0.0000	0.0067
1666	0.3607	0.0005	0.0000	0.6143	0.1786	0.0076	0.0171	0.3675	0.0185	0.0987	0.0001	0.0000	0.0658	0.0000	0.8644
	0.0232	–	0.0000	0.0119	0.0067	0.0029	0.0023	0.0148	0.0036	0.0092	0.0001	0.0000	0.0112	0.0000	0.0060
1768	0.0093	0.0009	0.2678	0.0293	0.0143	0.7320	0.0198	0.0000	0.0912	0.0172	0.0000	0.0000	0.0000	0.0002	0.2415
	0.0036	–	0.0173	0.0060	0.0024	0.0128	0.0037	0.0000	0.0080	0.0037	0.0000	0.0000	0.0000	0.0002	0.0108
1464	0.0087	0.0009	0.0455	0.0133	0.4563	0.0731	0.0112	0.1357	0.2643	0.0314	0.0001	0.0000	0.0000	0.0000	0.0081
	0.0022	–	0.0076	0.0021	0.0071	0.0054	0.0018	0.0142	0.0084	0.0029	0.0001	0.0000	0.0000	0.0000	0.0053

Table S3 to be continued

1363	0. 0925	0. 0009	0. 0007	0. 3446	0. 0019	0. 5500	0. 0000	0. 0765	0. 0001	0. 0034	0. 0002	0. 0000	0. 0096	0. 0000	0. 0045
	0. 0116	–	0. 0005	0. 0181	–	0. 0244	0. 0000	0. 0088	0. 0001	0. 0011	0. 0002	0. 0000	0. 0033	0. 0000	–
652	0. 0000	0. 0021	0. 0413	0. 3722	0. 0000	0. 5711	0. 0010	0. 0000	0. 0180	0. 0054	0. 0087	0. 0034	0. 0000	0. 0020	0. 1674
	0. 0000	0. 0007	0. 0082	0. 0203	0. 0000	0. 0238	0. 0005	0. 0000	0. 0053	0. 0022	0. 0024	0. 0021	0. 0000	0. 0010	0. 0109
1869	0. 3855	0. 0028	0. 2485	0. 0284	0. 1221	0. 0198	0. 0000	0. 0000	0. 0038	0. 0141	0. 0000	0. 0000	0. 0205	0. 0000	0. 2633
	0. 0344	–	0. 0148	0. 0025	0. 0076	0. 0046	0. 0000	0. 0000	0. 0009	–	0. 0000	0. 0000	0. 0086	0. 0000	0. 0181
1060	0. 0339	0. 0647	0. 0142	0. 0000	0. 0003	0. 0031	0. 0000	0. 0000	0. 0028	0. 0047	0. 0000	0. 0000	0. 0258	0. 0000	0. 0056
	0. 0111	0. 0083	0. 0021	0. 0000	–	0. 0017	0. 0000	0. 0000	0. 0009	–	0. 0000	0. 0000	0. 0079	0. 0000	–
1162	0. 1038	0. 0211	0. 2149	0. 1180	0. 0338	0. 0078	0. 0387	0. 0015	0. 0000	0. 0079	0. 0000	0. 0165	0. 0887	0. 0000	0. 0542
	0. 0174	0. 0030	0. 0227	0. 0162	0. 0048	0. 0025	0. 0093	0. 0009	0. 0000	0. 0023	0. 0000	0. 0057	0. 0104	0. 0000	0. 0031
858	0. 0615	0. 0754	0. 0177	0. 0000	0. 0045	0. 3664	0. 0000	0. 0020	0. 0031	0. 0003	0. 0000	0. 0000	0. 0000	0. 0000	0. 1783
	0. 0131	0. 0046	0. 0038	–	–	0. 0167	0. 0000	0. 0009	0. 0010	–	0. 0000	0. 0000	0. 0000	0. 0000	0. 0134
248	0. 0882	0. 0041	0. 0431	0. 0018	0. 2417	0. 0000	0. 0000	0. 0006	0. 0180	0. 0016	0. 0000	0. 0000	0. 0000	0. 0000	0. 0443
	0. 0117	0. 0013	0. 0110	0. 0007	0. 0092	0. 0000	0. 0000	0. 0006	0. 0038	–	0. 0000	0. 0000	0. 0000	0. 0000	0. 0038
